# Feasibility and acceptability of group music therapy vs wait-list control for treatment of patients with long-term depression (the SYNCHRONY trial): study protocol for a randomised controlled trial

**DOI:** 10.1186/s13063-017-1893-8

**Published:** 2017-03-29

**Authors:** Catherine Elizabeth Carr, Julian O’Kelly, Stephen Sandford, Stefan Priebe

**Affiliations:** 1 Unit for Social and Community Psychiatry, WHO Collaborating Centre for Mental Health Services Development, Queen Mary University of London, Newham Centre for Mental Health, Glen Road, London, E13 8SP UK; 2 East London NHS Foundation Trust, 9 Alie Street, Tower Hamlets, London, E1 8DE UK

**Keywords:** Group music therapy, Chronic depression, Songwriting, Feasibility, Randomised controlled trial, Nested process evaluation

## Abstract

**Background:**

Depression is of significant global concern. Despite a range of effective treatment options it is estimated that around one in five diagnosed with an acute depressive episode continue to experience enduring symptoms for more than 2 years. There is evidence for effectiveness of individual music therapy for depression. However, no studies have as yet looked at a group intervention within an NHS context. This study aims to assess the feasibility of conducting a randomised controlled trial of group music therapy for patients with long-term depression (symptom durations of 1 year or longer) within the community.

**Methods:**

This is a single-centre randomised controlled feasibility trial of group music therapy versus wait-list control with a nested process evaluation. Thirty participants will be randomised with unbalanced allocation (20 to receive the intervention immediately, 10 as wait-list controls). Group music therapy will be offered three times per week in a community centre with a focus on songwriting. Data will be collected post-intervention, 3 and 6 months after the intervention finishes. We will examine the feasibility of recruitment processes including identifying the number of eligible participants, participation and retention rates and the intervention in terms of testing components, measuring adherence and estimation of the likely intervention effect. A nested process evaluation will consist of treatment fidelity analysis, exploratory analysis of process measures and end-of-participation interviews with participants and referring staff.

**Discussion:**

Whilst group music therapy is an option in some community mental health settings, this will be the first study to examine group music therapy for this particular patient group. We will assess symptoms of depression, acceptability of the intervention and quality of life. We anticipate potential challenges in the recruitment and retention of participants. It is unclear whether offering the intervention three times per week will be acceptable to participants, particularly given participants’ enduring symptoms and impact upon motivation. This study will provide data to inform both development of the intervention and to assess and inform the design of a full trial.

**Trial registration:**

ISRCTN.com, ISRCTN18164037. Registered on 26 September 2016.

**Electronic supplementary material:**

The online version of this article (doi:10.1186/s13063-017-1893-8) contains supplementary material, which is available to authorized users.

## Background

Depression is of significant global concern. The World Health Organisation has predicted that by 2020 it will become the second leading contributor to the global burden of disease [1], and accounts for around 40% of suicides by people receiving treatment for mental illness in the UK [2]. It is estimated that between 8 and 12% of the UK population experience depression in any year [3], whilst around one in five diagnosed with an acute depressive episode goes on to develop chronic depression [1]. Chronic or persistent depression is diagnosed when the symptoms of depression endure for 2 years or longer. The severity and course of symptoms may vary, from milder symptoms of dysthymia to chronic major depression [3]. For this specific patient group, the median duration can be anything between 5 and 20 years [4, 5] and treatment is particularly difficult. Frequent relapses can lead to pessimism and demoralisation of both patient and professional [5], leading in turn to lack of compliance or ‘giving up’ on treatment. Overall, chronicity of depression is associated with increased healthcare costs through greater use of services and rates of hospitalisation [2, 6, 7].

Whilst the definition of chronic or persistent depression is marked by symptoms lasting 2 years or longer, durations of 1 year or longer are clinically relevant and may be indicative of a chronic course [8, 9]. It is estimated that around 40% of patients with chronic depression fulfil criteria for treatment resistance, although further evidence suggests that many may not have received adequate or recommended treatment [10]. Given that treatment resistance may be identified as soon as 6 months after diagnosis (or after two trials of antidepressant drugs [11]), symptoms enduring for 1 year or longer may be considered as an indicator of both potential chronicity and need for further intervention. There may also be clinical benefit to offering intervention at a point before symptoms have become long-standing. To avoid confusion with defined criteria for chronic or persistent depression, this study uses the term ‘long-term depression’ to define patients with symptoms of depression that have lasted 1 year or longer.

### Current treatments for long-term depression

Current recommended interventions for long-term depression are a mixture of either pharmacological or talking-based approaches, with mixed evidence on their combined use. Earlier reviews have indicated that both pharmacotherapy [12, 13] and psychotherapy [14] are effective treatments for chronic depression. The effects appear to be maximised when used in combination, although around 18 sessions of psychotherapy may be necessary in order to see clinical effects [15]. A more recent review found limited evidence for their use in combination [16] but suggested psychotherapy might have a continued role in promoting and maintaining treatment adherence, given that patient preferences are often for psychotherapy over medication and that they achieve wider clinical benefits (such as improving coping strategies and quality of life). Two further reviews [17, 18] point towards an approach for use of both in combination, leading the European Psychiatric Association to recommend combined treatment with a personalised approach based on patient preferences and needs [19]. Based on current evidence, psychotherapeutic interventions that target interpersonal problems (such as the cognitive behavioural analysis system of psychotherapy (CBASP) and interpersonal psychotherapy (IPT)) appear to be indicated in addressing symptoms of chronic depression, whilst further studies indicate that long-term psychoanalytic psychotherapy may also improve long-term outcomes in treatment-resistant depression [20].

Childhood adversity and maltreatment such as abuse and problems with the family environment have been linked to the development and chronicity of major depressive disorder [9, 19, 21–29]. A review of risk factors for chronic depression suggested that along with a younger age of onset and family history of mood disorder, problems within the social environment (such as low social integration, low social support and negative social interaction) were associated with chronicity of symptoms [4]. A group approach may therefore be of utility in addressing social integration, interaction and providing emotional and social support, and have potentially greater cost-effectiveness.

### Group music therapy: evidence to date

Music therapy is a complex intervention in that it utilises a range of components to promote health. Such components include a therapeutic relationship, a range of active and receptive musical activities and verbal reflection. These are provided flexibly in response to the individual or group and are often led by the patient. Activities include improvisation, songwriting and use of pre-known music, with opportunities for verbal reflection. Within the UK, music therapists are regulated by the Health and Care Professions Council (HCPC) and must have completed approved training to Masters level.

There is evidence to suggest the effectiveness of music therapy for a range of mental health problems including depression [30], schizophrenia [31, 32] and severe mental illness [33, 34]. A meta-analysis by Gold et al., has identified a dose-effect response, whereby symptom improvement is associated with the number of sessions received [34]. The analysis suggested that around 4 sessions would be required to have a small effect on depressive symptoms, 10 for a medium effect and 16 for a large effect. For global functioning this was estimated at 3 sessions for a small effect to 51 sessions for a large effect and for general symptoms, 10 sessions for a small effect to 39 sessions for a large effect. The impact of session frequency and duration is less clear and within this meta-analysis [34], varied between one and six times per week over durations of 1 to 6 months.

A Cochrane review of music therapy for depression included only five studies, three of which were in elderly populations, one of which was in children in schools and one of which was in adult psychiatric inpatients [35]. As such, these were too heterogeneous to compare in a meta-analysis. Four of these studies reported a greater reduction in symptoms of depression compared to standard care, whilst one study using music therapy as an active control found no significant change in comparison to standard care. The authors noted low drop-out rates leading the authors to conclude that whilst the intervention appears to have good acceptability, conclusions could not be drawn on its effectiveness [35].

Two further randomised controlled trials have since been published: Erkkilä et al. [30] sought to determine the efficacy of individual music therapy for depression compared to standard care. Music therapy consisted of free improvisation and discussion informed by psychodynamic theory, for 60 minutes, twice a week for 10 weeks. At the 3-month follow up, patients in the music therapy group had greater improvement in depression, anxiety and general functioning compared to the control group. Improvements were also seen in alexithymia and quality of life and were sustained at the 6-month follow up, but these were not statistically significant when compared to the control group. The second trial [36] looked at the effect of group improvisational music therapy on depression in adolescents and adults with substance abuse. Twenty-four patients were randomly allocated to 12 sessions of music therapy plus standard care or standard care alone. At the end of the 12 sessions, there were significant differences between groups in observer-rated depression but not self-rated depression, with greater improvements in the music therapy group.

Wider randomised controlled trials of music therapy in mental healthcare suggest it may be particularly effective in encouraging people with low motivation and serious mental disorders to attend and engage in therapy [33, 37, 38]. Notably, a study of group music therapy in the community demonstrated improved quality of life in patients with severe mental illnesses [37]. It may be that nonverbal aspects of communication may make it easier or more acceptable for people who find it difficult to utilise talking therapies and may help them to re-engage with these at a later point, as seen in a study of music therapy for post-traumatic stress disorder [38].

### Theoretical basis of the intervention

A core problem when working with chronic depression is demoralisation of both patient and professional, leading in turn to lack of compliance or ‘giving up’ on treatment [5]. Group music therapy may offer a very different therapeutic encounter compared to current treatments and has the potential to address intrapersonal and interpersonal domains and promote confidence and participation at a community level. The experience of making music provides an opportunity for nonverbal expression and communication, strong shared group experiences, rehearsal of different ways of relating and opportunities to have a sense of achievement, ‘perform’ or share different, non-illness parts of themselves [39]. A positive experience within a community-based music therapy group may then place the person in contact with their musical and inner psychological ‘resources’ [40], which may provide opportunities to build inner resources for coping and resilience and promote hope, and can be linked to wider theories of personal recovery in mental illness [41, 42].

Long-term depression can be understood from a variety of biological, neurological, psychological, relational and social perspectives. We believe that Seligman’s model of learned helplessness provides the clearest starting point for understanding how music therapy might help long-term depression [43]. This theory suggests that with ongoing aversive events, a person may perceive increasing lack of control and helplessness. Such repeated events lead to a downward spiral of lessening confidence and sense of control of the situation, reducing motivation, retreating from the environment (thus impacting upon wider social relationships) and thus impacting upon mood and wider functioning. In long-term depression such experiences are manifested in the prolonged low mood, low feelings of self-worth, hopelessness, lack of energy, irritability and insomnia. These symptoms then have a wider impact upon the individual’s emotions, cognitions, social life and wider functioning.

Erkkilä and colleagues [44] use a related theory of withdrawal motivation [45] to explain, within the framework of psychodynamic theory, how such symptoms impact upon a person’s ability to create and symbolize - processes that both have the ability to transform a feeling state, or connect meaningfully with another person. The use of free improvisation (a form of active music-making) is assumed by music therapists to be something that can be accessed by all as a means of expressing internal states, creating and connecting with others. The nonverbal interactions that occur within such music-making enable a different experience of communicating and relating to others, in a medium that has been shown to be motivating and stimulating for people who otherwise find it difficult to engage [33]. Such experiences may enable opportunities for more positive social experiences than those experienced verbally, and opportunities to then verbally process these with other members of the group. The musical attunement facilitated by the music therapists when making music may help participants to experience nonverbal social contact and closeness, and address feelings of social isolation [46]. Such a process is implicated in building initial therapeutic trust, which again, has been noted to be of importance for this clinical group [44].

Consultation with patient and public groups at the study inception suggested that the intervention should be community-based with a strong sense of structure and musical ‘end-product’ such as recording or performance, as this would build self-esteem and foster feelings of achievement. Singing was frequently suggested as a more accessible way to make music. We have therefore incorporated a group music-therapy songwriting intervention developed in Australia [37, 47] alongside principles from psychodynamic improvisational music therapy [44] and resource-oriented music therapy [40, 48].

Studies of singing and songwriting suggest that group singing can most powerfully invoke positive emotions, feelings of connectedness to others and group bonding [49–52] and act as encouragement for people to return to and participate in groups [53]. Creating bespoke songs as a group has the added potential for participants to begin to find ways of verbally expressing their experiences and having this supported through active group music-making [44, 54–56].

Taken in combination, the evidence for music therapy to address symptoms of long-term depression is promising, but to date the intervention has not been specified or tested for its effectiveness within a UK National Health Service (NHS) context.

## Rationale for the current study

Before evaluating the effectiveness of an intervention, it is important to assess whether implementation of the trial methodology is feasible. This is particularly relevant in complex interventions and settings [57–60].

### Feasibility of the intervention

Whilst music therapy is commonly provided in community mental health settings, this is the first time the intervention has been tailored for this particular diagnostic group with an emphasis on songwriting [37, 55]. It will therefore be important to assess whether the intervention is delivered as described in practice and to examine the role of each component in relation to outcomes. It will also be necessary to examine the acceptability of the intervention (both in terms of how it is provided and its individual components) to patients and music therapists, as this will have a bearing on whether the intervention can feasibly be delivered as described. Information on the intervention in this pilot will be used to refine and where necessary, adapt the current approach to ensure acceptability, adherence and treatment fidelity.

### Feasibility of the research design

To our knowledge a randomised controlled trial of group music therapy has not been conducted within this set of NHS services before. The feasibility of our proposed methods for identifying, recruiting and retaining participants with chronic depression from primary care and secondary care NHS services is not yet known. Piloting on a small scale will enable us to determine both the feasibility of our proposed processes and the resources and approaches required [61].

### Estimation and appropriateness of outcomes

We expect to see changes in a range of clinical and social outcomes. However, we do not yet know which measures might be most appropriate in terms of acceptability of completion and variability of outcome. Our proposed primary outcome is symptoms of depression, measured by an observer-rated scale (Montgomery-Åsberg depression rating scale [62]). We will also measure potential changes in self-rated depression, social functioning, psychological distress, self-esteem, self-efficacy, mood, relationships, satisfaction with treatment, work and social adjustment, quality of life and service use. We will examine the acceptability and provide descriptive statistics and estimates of effect of the proposed outcome measures to inform the design of a future randomised controlled trial. As we are providing a group intervention, we also plan to estimate the intra-cluster correlation coefficient to take this into account in conjunction with future sample size calculations. To aid estimation of the cost of the intervention, we plan to conduct a preliminary economic analysis to estimate the cost of services received by the intervention and control groups.

### Design considerations

For comparison we have chosen to use a wait-list control. This will enable us to compare the intervention to treatment as usual, with the option for those in the control group to receive group music therapy at the study end.

We propose offering three sessions per week to participants for a number of reasons. First, in a previous study in a similar environment [37] there were high rates of attrition in the once-per-week group. Our previous research within an acute inpatient environment demonstrated that offering an increased frequency was acceptable, of therapeutic benefit and increased the chances of attending a greater number of music therapy sessions [53]. Second, given the potential difficulties in motivation and attending sessions, the increased frequency may help to build momentum and keep attendance going. Greater frequency also means it may be possible to develop a greater stability of group membership, which will help to foster trust and commitment in the group and, in the case of ongoing songwriting and performance work, provide opportunities to progress these longer-term activities. In total, 42 sessions are available to participants, which may enable us to detect a large effect on depressive symptoms, and medium-to-large effects on general symptoms [34].

## Methods

### Design

This is a single-centre, randomized controlled feasibility trial of group music therapy versus wait-list control with post-intervention, 3-month and 6-month post-intervention follow up and nested process evaluation.

### Aims and objectives

The study aims to pilot group music therapy for patients with long-term depression and assess the feasibility of conducting a larger randomised controlled trial. The results will inform the design of future studies, the refinement of the manual and training of music therapists. Should the trial be found feasible, a protocol will be produced for a full-scale randomised controlled trial.

The objectives are to:

1. Assess the acceptability of the methodology to professionals and patients
2. Assess the feasibility of recruitment processes
3. Identify the number of eligible participants, participant rates and retention rates
4. Assess the researcher time and costs per participant
5. Assess the appropriateness of outcome measures, data on the variability of the outcome for sample size calculation, an estimate of the control group mean to ensure change is feasible and the intra-cluster correlation coefficient
6. Assess the intervention in terms of testing components, measuring adherence and estimating the likely intervention effect
7. Assess the exact cost of delivery of group music therapy and the services received

### Setting and participants

This is a single-site study with group music therapy provided in a community location by East London NHS Foundation Trust music therapists. Thirty participants will be recruited from secondary care Community Mental Health Teams (CMHTs), Improving Access to Psychological Therapies (IAPT) services and referrals from Participant Identification Centres (PICs) within primary care general practice (GP) services.

Patients are eligible for study entry if they meet the following criteria:

- Confirmed diagnosis of depression (ICD10 F31-39) including post-schizophrenic depression (ICD10 F20.4) and prolonged depressive reaction (ICD10 F43.21)
- Receiving pharmacological and/or psychological treatment for 12 months or longer
- Aged 18 years or above
- Capacity to give informed consent

Patients will not be eligible if any of the following are present:

- Diagnosis of organic mental disorder (ICD10 F00-09)
- Bipolar affective disorder - current manic episode (ICD10 F30, F31.0, F31.2, F31.6, F31.70-F31.74)
- No capacity to give informed consent
- Risk of suicide necessitating hospitalisation

### Participant identification and recruitment process

**Fig. 1:**
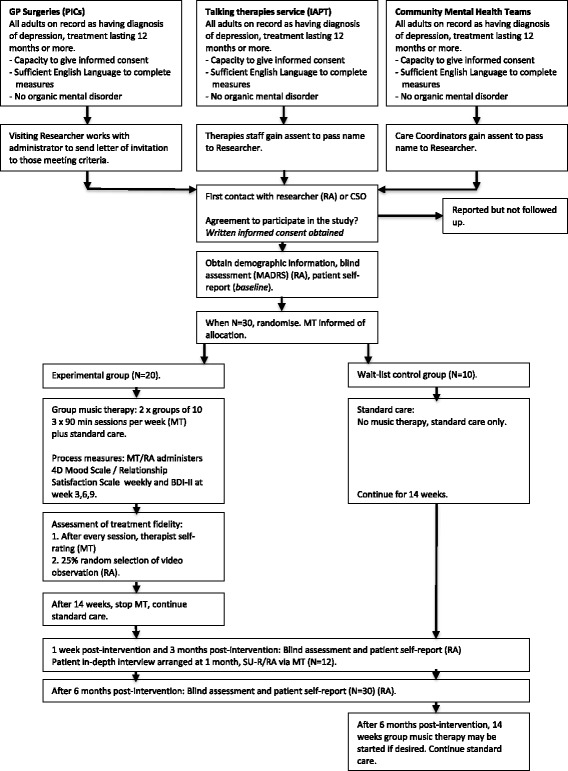
Flow diagram of the SYNCHRONY study to assess the feasibility of group music therapy for chronic depression. *BDI-II* Beck Depression Inventory-II, *CSO* Clinical Studies Officers, *GP* general practice, *IAPT* Improving Access to Psychological Therapies, *MADRS* Montgomery-Åsberg Depression Rating Scale, *MT* Music Therapists, *PIC*s Participant Identification Centres, *RA* Research Assistant, *SU-R* Service-User Researcher

Participant identification, recruitment and follow-up procedures are shown in Fig. 1. Recruitment will commence 8 weeks before the first scheduled music therapy group. Patient identification will be made by a member of the patient’s existing clinical care team in the following ways:

1. Primary care PICs: GP surgeries. All adults on record as meeting the inclusion criteria will be identified by an administrator working within the service. The researcher will provide a letter of invitation which will be sent out with an information sheet. Those expressing interest will be invited to speak with the researcher for further information.
2. Talking therapies (IAPT) service and CMHTs. Patients meeting the inclusion criteria will be informed of the study and provided with an information sheet by the healthcare professional overseeing their care. Assent will be gained for the professional to put the patient in touch with the researcher. Where possible, a researcher will be based on site to speak with patients to minimise further additional visits.

Interested participants will meet with a member of the research team to go through the study information and answer any questions. Inclusion and exclusion criteria will be confirmed along with assessment of mental capacity. Written informed consent will be taken a minimum of 24 hours later along with baseline assessments. Participants are informed they are free to withdraw at any time without giving reasons and without prejudicing any further treatment.

### Intervention

The intervention has been described in full in a manual developed for the purposes of this study and has been informed by existing manuals for group songwriting [44], individual psychodynamic music therapy for depression [30, 47] and principles of resource-oriented music therapy [48]. The intervention was developed through focus group discussions with music therapists, psychologists and service users with a diagnosis of depression and was finalised in team discussions with music therapists and Heads of Arts Therapies involved in the study. The Template for Intervention Description and Replication [63] and Standard Protocol Items: Recommendations for Interventional Trials (SPIRIT) [64] checklists are attached as Additional files 1 and 2. The intervention manual and full study protocol are available on request from the authors.

Group music therapy will be provided three times per week over 14 weeks by two HCPC-registered music therapists. Each session will last 90 minutes and will comprise warm-up activities moving to sharing of pre-known songs and improvisation. Therapists will move from improvisation into songwriting, which will then be the main focus for the remainder of the session. Opportunities will be offered after each activity for verbal reflection. The last 15 minutes will be dedicated to reviewing the session and may include playing through a final piece of music.

The intervention will be provided in a community centre within the locality of Newham. The community centre is a non-NHS site and offers facilities for additional social contact such as a café and wider non-medical community groups. Music therapists will receive weekly clinical supervision from the chief investigator.

### Wait-list control

The wait-list control group will receive treatment as usual whilst study measures are completed and will then be eligible to receive group music therapy once the 6-month follow up has been completed. Receipt of services will be assessed throughout this time in both arms to enable an initial description of the types of services received and associated cost.

### Criteria for discontinuation

Participants will be withdrawn from the intervention if the participant becomes too unwell to continue due to loss of capacity to consent to group attendance, if the patient has a level of risk assessed by the clinical team to require hospitalisation or if music therapists and the clinical team determine that the patient’s current mental state, behaviour or risk to self or others requires discontinuation of the intervention. Reasons for withdrawal will be documented as part of feasibility measures.

### Randomisation

Randomisation will occur once all 30 participants have been recruited and baseline measures have been completed. In order to obtain sufficient data on the intervention, we will utilise simple block randomisation with unbalanced allocation so that 20 participants receive the intervention and 10 are allocated to the wait-list control group. A researcher independent of the study team will perform the randomisation using the Experimental Design Generator and Randomiser (EDGAR-II) [65] and inform the music therapists and unblinded member of the study team of the allocation by email. The music therapists will then inform participants of their allocation and provide additional support if required.

### Blinding

Blinding of participants and therapists is not possible due to the study intervention and timing of assessments. Researchers conducting follow-up assessments will be blind to participant allocation. An independent statistician will conduct the initial analysis of the main outcomes Due to the unbalanced design it will not be possible to blind the statistician to the treatment allocation.

### Outcome measures and endpoints

Our primary interest is in the feasibility of the trial design and intervention. A summary of the main feasibility outcomes, success criteria and timing is shown in Table 1. Proposed clinical outcomes are:

**Table 1:** Methods and timing for assessing, recording and analysing outcome parameters

Outcome	Method	Success criteria				Timing
		Stop	Continue, modify protocol	Continue without modification but monitor closely	Continue without modifications	
Acceptability of methodology	Recruitment and retention rates as below					End of recruitment (week 8)
	Compliance	Mean attendance <10 sessions	Mean attendance <14 sessions	Mean attendance 14 sessions	Mean attendance 14+ sessions	End of intervention (week 22)
	End interviews	Unfavourable views, serious concerns	Unfavourable views, suggestions for modification	Favourable views, suggestions for modification	Favourable views, no concerns	1 month post-intervention (week 26)
Feasibility of recruitment processes	Screening rates	Identify <50 potentially eligible subjects	Identify <100 potentially eligible subjects	Identify 100–128 potentially eligible subjects	Identify >128 potentially eligible subjects	End of recruitment
	Recruitment rates	Recruit <50% of sample size	N <25 in 8wks, <5% per week	*N* = 25–30 in 8wks, <13% per week	*N* = 30 in 8wks, 13% per week or greater	End of recruitment
	Participation rates	Participation rate <5%	Participation rate 5–15%	Participation rate 15–25%	Participation rate 25% or greater	6 months post-intervention
	Retention rates	Attrition >75%	Attrition 50–75%	Attrition 30 − 50%	Attrition <30%	6 months post-intervention
	End interviews	N/A	Major suggestions to improve recruitment processes	Minor suggestions to improve recruitment processes	No suggestions to improve expressed	1 month post-intervention (week 26)
Identify number of eligible participants, participant rates and retention rates	Number identified by HCPs	<50 identified	50–100 identified	100–128 identified	>128 potentially eligible identified	End of recruitment
	Number expressing interest	<30 express interest	30–40 express interest	40–60 express interest	>60 express interest	End of recruitment
	Number providing consent	<15 provide consent	15–25 provide consent	25–30 provide consent	30 provide consent	End of recruitment post-intervention, 3 and 6 months post-intervention
	Number lost to follow up	Attrition >75%	Attrition 50–75%	Attrition 30–50%	Attrition <30%	
Researcher time and costs per participant	Researcher diary	N/A	Researcher time exceeds allocated time requiring additional study support	Researcher time and cost only just covers time required	Researcher time and cost fully covers time required	6 months post-intervention
Appropriate outcome measures	Variability of outcome Estimate of control mean and SD of change	No difference or clinically important difference favouring control detected based on confidence limits	Difference cannot be detected based on confidence limits but data suggest improvement favouring intervention	Difference can be detected based on confidence limits	Clinically important difference can be detected based on confidence limits	End of intervention
Intervention components	Therapist adherence	Adherence <50%	Adherence <50%	Adherence 50–75%	Adherence >75%	End of intervention
	End interviews	Serious concerns expressed regarding intervention	Major suggestions to adapt intervention	Minor suggestions to adapt intervention	No concerns or suggestions to adapt intervention	
Intervention adherence	Therapist self-rated adherence Video-rated adherence	Adherence <25%	Adherence 25–50%	Adherence 50–75%	Adherence >75%	End of intervention
Estimate of cost of intervention and services received	Therapist time CSRI	Cost significantly greater than usual care, no potential to modify intervention, no indication of benefits	Cost is greater than usual care - intervention may be modified, but outcomes suggest some benefits	Cost is greater than usual care but outcomes strongly suggest benefits	Cost is equivalent to or slightly greater than usual care, outcomes strongly suggest benefits	6 months post-intervention

*HCPs* healthcare professionals, *N/A* not applicable, *CSRI* Client Services Receipt Inventory

Observer rated:

1. Symptoms of depression as measured by the Montgomery-Åsberg Depression Rating Scale (MADRS) [62, 66, 67]
2. Social functioning as measured by the life skills profile (LSP) [68]
3. Level of hospitalisation as measured by admission, length of stay and number of readmissions

Self-report:

4. Self-reported symptoms of depression as measured by the Beck Depression Inventory II (BDI-II) [69–71]
5. Psychological distress as measured by the Brief Symptom Inventory (BSI) [72]
6. Self-esteem as measured by the Rosenberg Self-Esteem Scale (RSES) [73–75]
7. Perceived self-efficacy as measured by the General Perceived Self-Efficacy Scale (GPSES) [76–78]
8. Satisfaction with treatment in community services as measured by the Client Satisfaction Questionnaire (CSQ) [79]
9. Work and social adjustment as measured by the Work and Social Adjustment Scale (WSAS) [80]
10. Quality of life as measured by the Manchester Short Quality of Life Scale (MANSA) [81, 82]
11. Receipt of services as measured by the Client Services Receipt Inventory (CSRI) [83, 84]

Our proposed clinical endpoint is the week post-intervention, with secondary endpoints at 3 and 6 months post-intervention. Process measures of attendance, self-reported depression symptoms (BDI-II [69]) at weeks 3, 6 and 9 and weekly mood pre-session and post-session (Dispositional Mood Scale (DMS) [85, 86]) and group relationships (Relationship Satisfaction Scale (RSS) [87]) will be measured.

### Data collection

**Fig. 2:**
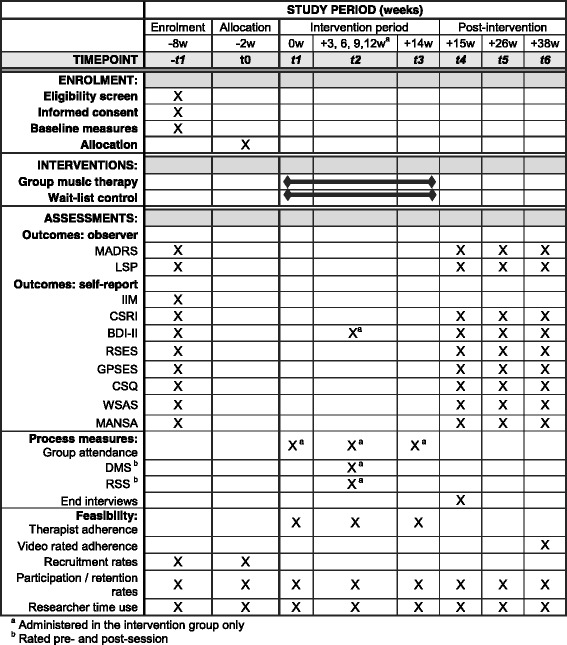
Standard Protocol Items: Recommendation for Interventional Trials (SPIRIT) diagram of assessments at enrolment, allocation, 3-weekly sessions, post-intervention, 3-month and 6-month time points. ^a^Administered in the intervention group only. ^b^Rated pre-session and post-session. *BDI-II* Beck Depression Inventory-II, *CSQ* Client Satisfaction Questionnaire, *CSRI* Client Services Receipt Inventory, *DMS* Dimensional Mood Scale, *GPSES* General Perceived Self-efficacy Scale, *IIM* Interest in Music Scale [95], *LSP* Life Skills Profile, *MADRS* Montgomery-Åsberg Depression Rating Scale, *MANSA* Manchester Short Quality of Life Scale, *RSES* Rosenberg Self-esteem Scale, *RSS* Relationship Satisfaction Scale, *WSAS* Work and Social Adjustment Scale

A schedule of data collection is shown in the SPIRIT diagram in Fig. 2 [64]. Inter-rater training was conducted for observer-rated measures prior to recruitment. Inter-rater reliability between researchers for the MADRS was high (ICC = .995 (*p* < .001), 95% CI .987 to .999).

Baseline assessments will occur as soon as possible after informed consent is obtained. Follow-up visits will be arranged in the week following the end of the intervention, and at 3 and 6 months after the intervention end. Participants in the intervention arm will each receive a payment of £10 to contribute towards travel costs and participants in the wait-list control group will each receive £15 in the form of shopping vouchers. Participants will be eligible to receive assistance with the cost of transport to and from therapy sessions if they currently do not receive such help.

Researchers conducting blind follow-up assessments will enter the data onto paper case report forms designed for each assessment. These will be transferred into an electronic database and double-entered. Limits are set on data values to be entered to ensure only valid entries are permitted.

### Nested process evaluation

We will conduct a process evaluation with the following aims:

1. To understand exactly how the intervention was delivered in practice (treatment fidelity analysis)
2. Describe processes of attendance and hypothesised process factors of self-reported depression, mood and group relationships from week to week
3. Understand subjective experiences and attributions for change of the intervention from the perspective of patients, music therapists and referring staff
4. Compare reported quantitative and qualitative processes against the proposed logic model and to revise accordingly

The process evaluation will employ an embedded mixed methods design and will consist of data collection of video data of the intervention itself, client self-reported measures (weekly quantitative process measures of mood pre-session and post-session (DMS [86]), group relationships pre-session and post-session (RSS) [87]) and qualitative end interviews 1 month post-intervention. The interview is optional for service users but we aim to conduct a minimum of 12 interviews.

Quantitative analysis will provide a descriptive analysis of the course of weekly process measures. We will descriptively explore whether there are any differences between compliant and non-compliant attenders, responders and non-responders and whether any socio-demographic and clinical characteristics are associated with outcomes.

Qualitative evaluation will comprise end-of-study interviews with 12 participants, their referring staff and music therapists. Whilst interviews will have a component to focus upon experiences of the research and views on the design, a second part will ask for views on the experience of the group music therapy itself. These questions are based upon a pre-existing semi-structured interview (Client Change Interview [88, 89]) to elicit clients’ views of and attributions for change. We have adapted this interview for music therapists and referring clinicians to also reflect upon possible observed changes in the participants who they have worked with and their views of the intervention.

Interviews will be transcribed and imported into NVivo qualitative analysis software and read a number of times by members of the research team. Members will individually code 25% of the interviews and then meet to decide upon a preliminary thematic frame. As interviews will be asking about individual experiences, coding will take a phenomenological perspective and seek to retain the essence of individual narratives. The team will continue to code and meet regularly to discuss the adequacy of the frame in reflecting individual experiences. Once a final thematic frame is agreed, we will then explore relationships between the themes identified in the data and quantitative findings. Quantitative and qualitative data will be compared against the hypothesised logic model for our intervention. We will then refine and revise the logic model based upon these findings.

### Sample size

This study aims to assess the feasibility of recruitment processes including the number of eligible participants and participation/retention rates. Papers considering sample size for feasibility and pilot studies suggest the inclusion of upwards of 24–50 participants [59, 90, 91]. The present study aims to recruit 30 patients to participate in three groups of 10 patients each. In order to obtain sufficient data on the intervention, randomisation will be imbalanced so that 20 are randomised to the treatment arm and 10 to the wait-list control group.

Studies of wider psychosocial and psychotherapeutic interventions for chronic depression show participation rates in the range of 25–33% of eligible patients consenting to participate in the study [92–94]. With a sample size of 30, we will be able to estimate a participation rate of 25% to within a 95% confidence interval of +/-15%. Assuming that one fifth of patients accessing primary care for depression will have developed chronic depression, we estimate approximately 1300 patients within primary care will be eligible for this study. We estimate each practice will have around 100 patients with depression, of which around 20 will have enduring symptoms. Within the secondary care sites in which we plan to recruit, there are currently around 1960 patients with a diagnosis of depression. Assuming that one fifth will have developed chronic depression [1], we estimate approximately 392 patients within secondary care will be eligible for this study. Based on a participation rate of 25%, we plan to approach 128 patients with the aim of recruiting 4 per week, over 8 weeks.

### Statistical analysis

#### Feasibility

As this is a feasibility study, the main statistical reporting will comprise descriptive statistics and calculation of effect sizes. We will assess:

• Acceptability of methodology to professionals and patients through interviews via recruitment rates, compliance, end interviews      
• Feasibility of recruitment processes
• Deviation around specific post-intervention, and 3-month and 6 month post-intervention follow-up assessment dates for actual data collection
• Number of eligible participants, participation rates and retention rates
• Researcher time and costs per participant
• Appropriateness of outcome measures

Descriptive analyses will establish recruitment and drop-out rates and the distribution of baseline characteristics and all outcomes post-intervention, and 3 months and 6 months post-intervention. The research team will reflect upon the representativeness of the demographics in terms of the general population of patients that could potentially be useful in assessing the intervention.

Recruitment and drop-out rates will be monitored on a monthly basis allowing for examination and reporting of patterns and whether there are any obvious trends. This may inform the choice of the primary outcome for a full-scale trial allowing for drop-outs in a sample-size calculation.

#### Clinical outcomes

This study will employ intention-to-treat analysis. Data from all subjects will be included in the analysis as randomised. Adverse events will be reported by sub-groups of those who were currently receiving the intervention and those who were not.

We will first calculate a mean value of the outcome for each unit and present them with confidence intervals. Outcomes will be compared between intervention and control groups using linear regression models, adjusting for the baseline score of the given outcome. The confidence limits of each treatment effect and knowledge of the clinically important difference (where this is available) will be used to determine whether clinically important differences are ruled out by these confidence limits. The intra-cluster correlation coefficient will be calculated. Due to the small sample size, this estimate is likely to be imprecise, therefore we will also use estimates in the literature from similar studies for comparison to inform the design of any future studies. We will descriptively explore in sub-group analyses whether there are any differences between compliant and non-compliant attenders, responders and non-responders and whether any socio-demographic and clinical characteristics are associated with outcomes. As a preliminary economic evaluation, we will calculate the cost of delivery of music therapy and cost of services received by both groups using the CSRI [83]. This will be descriptive only to provide an estimate for future studies.

### Ethical and Health Research Authority approval

This study was reviewed and received a favourable ethical opinion by Wales Research Ethics Committee (REC) 2 on 13 September 2016 (Reference: 16/WA/0248) and host site approval was received from East London Foundation NHS Trust R&D office, via Noclor on 27.09.2016. All information collected will be kept confidential, stored securely and archived in accordance with the research governance policy of the sponsor. Participant anonymity will be retained by allocating a unique identification number for the trial and any identifiable information stored separately from this.

### Trial governance, monitoring and auditing

The Trial Management Group (TMG) consists of the chief investigator, study team and co-investigators and will provide overall management of the study including set-up, training, recruitment, promotion of the study and interpretation of results. Given the small and exploratory nature of the trial, no interim analysis is planned. Further to consultation with the study sponsor, the team considers that the nature of the intervention does not pose a significant risk to the study participants, and therefore we will not use a data monitoring committee. An Independent Advisory Panel (IAP) consisting of two independent members will monitor the conduct of the trial and meet four times over the course of the study to discuss research progress according to defined milestones, emerging difficulties and dissemination of results.

The chief investigator in liaison with the sponsor and the IAP has ultimate authority to halt the study or withdraw individual participants should concerns arise during the study. Adverse events will be monitored and recorded throughout. All serious adverse events will be reported to the sponsor and reported to the REC if assessed to be serious, related to the study and unexpected. Adherence to the protocol and any subsequent amendments will be supervised by the sponsor. Access to data will be granted to authorised representatives from the sponsor, host institution and the regulatory authorities, to permit trial-related monitoring, audits and inspections.

### Ancillary and post-study care

The music therapists and researchers will maintain contact with the participants’ clinical care teams throughout the study and immediately raise any areas of concern or need. Participants will be offered time to speak with music therapists at the end of the intervention and will be provided with information on music therapy groups and wider music-making opportunities available in the community.

## Discussion

The burden of depression upon society is well-recognised, both in human emotional and economic costs. For those suffering from chronic depression, options for treatment are few for a condition that can last approximately 5 to 20 years [4, 10]. Current evidence on music therapy in mental health suggests it may be an acceptable and tolerable intervention, which may offer a different experience from currently recommended treatment. However, there is no evidence on the effectiveness and cost-effectiveness of such an intervention within an NHS community context.

Building evidence for a complex intervention, such as music therapy, requires more than just an adequate trial design. The intervention requires a clear definition and model, and may require subsequent steps to refine this once examined in practice [57]. Wider research into music therapy has pointed to the importance of training of therapists in the research and intervention procedures [60]. This study will examine not only the practicalities of trial design (in terms of recruitment strategy, retention and outcome measurement) but will also provide a platform upon which the intervention can be specified for the particular needs of patients with long-term depression. Such findings can be immediately integrated and applied in wider music-therapy community mental health work.

Whilst we believe that the intervention may be appealing for those with long-term depression, we anticipate challenges in both maintaining attendance and retaining participants within the study. Measures to address this include assistance with transport costs and maintaining regular contact with participants. Whilst participants may have access to around 42 sessions, this will occur in a relatively short time frame of 14 weeks and it remains to be seen whether changes can be detected within our proposed measures.

### Trial status

The SYNCHRONY study began recruitment on 27 September 2016 with the intervention planned to start mid to late November 2016. The study will be a first step towards refining and testing the effectiveness and cost-effectiveness of group music therapy for long-term depression. Whilst the study is limited in its small scale and single locality, outcomes will contribute to a better understanding of how best to provide music therapy for this patient population and will enable realistic and feasible plans to be made for a future large-scale trial.

## Additional files

Additional file 1: Template for Intervention Description and Replication (TIDieR checklist). (PDF 390 kb)

Additional file 2: Standard Protocol Items: Recommendations for Interventional Trials (SPIRIT) checklist. (PDF 130 kb)

## Abbreviations

BDI-II: Beck Depression Inventory-II
BSI: Brief Symptom Inventory
CBASP: Cognitive behavioural analysis system of psychotherapy
CMHT: Community Mental Health Team
CSQ: Client Satisfaction Questionnaire
CSRI: Client Services Receipt Inventory
DMS: Dispositional Mood Scale
EDGAR-II: Experimental Design Generator and Randomisor - II
GP: General Practice
GPSES: General Perceived Self-efficacy Scale
HCPC: Health and Care Professions Council
IAP: Independent Advisory Panel
IAPT: Improving Access to Psychological Therapies
ICD-10: International Classification of Diseases, 10^th^ Edition
IPT: Interpersonal psychodynamic therapy
LSP: Life Skills Profile
MADRS: Montgomery-Åsberg Depression Rating Scale
MANSA: Manchester Quality of Life Scale
NHS: National Health Service
PIC: Participant Identification Centre
REC: Research Ethics Committee
RSES: Rosenberg Self-esteem Scale
RSS: Relationship Satisfaction Scale
SPIRIT: Standard Protocol Items: Recommendation for Interventional Trials
TIDier: Template for Intervention Description and Replication
TMG: Trial Management Group
UK: United Kingdom
WSAS: Work and Social Adjustment Scale

## References

[1] World Health Organisation. The World Health Report 2001 - Mental health: new understanding, new hope. World Health Organisation. 2001. http://www.who.int/whr/2001/en/. Accessed 08 Nov 2016.

[2] Centre for Suicide Prevention. National confidential inquiry into suicide and homicide by people with mental illness: making mental health care safer. Annual report and 20-year review. Healthcare Quality Improvement Partnership. 2016. www.Research.bmh.manchester.ac.uk/cmhs/research/centreforsuicideprevention/nci/reports/2016-report.pdf. Accessed 08 Nov 2016.

[3] Singleton N, Bumpstead R, O’Brien M, Lee A, Meltzer H. Psychiatric morbidity among adults living in private households 2000. Office for National Statistics. 2001. http://www.ons.gov.uk/ons/rel/psychiatric-morbidity/psychiatric-morbidity-among-adults-living-in-private-households/2000/psychiatric-morbidity-among-adults-living-in-private-households.pdf. Accessed 08 Nov 2016. 10.1080/0954026021000045967 12745312

[4] Hӧlzel L, Härter M, Reese C, Kriston L (2011). Risk factors for chronic depression−a systematic review. J Affect Disord.

[5] Koekkoek B, Van Meijel B, Hutschemaekers G (2008). Clinical problems in the long-term care of patients with chronic depression. J Adv Nurs.

[6] Gilmer WS, Trivedi MH, Rush AJ, Wisniewski SR, Luther J, Howland RH, Yohanna D, Khan A, Alpert J (2005). Factors associated with chronic depressive episodes a preliminary report from the STAR-D project. Acta Psychiatr Scand.

[7] Berndt ET, Koran LM, Finkelstein SN, Gelenberg AJ, Kornstein SG, Miller IW, Thase ME, Trapp GA, Keller MB (2000). Lost human capital from early-onset chronic depression. Am J Psychiatry.

[8] Uher J. Persistent depressive disorder, dysthymia and chronic depression: update on diagnosis, treatment. Psychiatric Times. 2014. http://www.psychiatrictimes.com/special-reports/persistent-depressive-disorder-dysthymia-and-chronic-depression. Accessed 08 Nov 2016.

[9] Brown GW, Craig TK, Harris TO (2008). Parental maltreatment and proximal risk factors using the Childhood Experience of Care and Abuse (CECA) instrument: a life-course study of adult chronic depression. J Affect Disord.

[10] Kocsis JH, Gelenberg AJ, Rothbaum B, Klein DN, Trivedi MH, Manber R, Keller MB, Howland RHY, Thase ME (2008). Chronic forms of major depression are still undertreated in the 21st century: systematic assessment of 801 patients presenting for treatment. J Affect Disord.

[11] Ruhe HG, van Rooijen G, Spijker J, Peeters FPML, Schene AH (2012). Staging methods for treatment resistant depression. A systematic review. J Affect Disord.

[12] Kocsis JH (2003). Pharmacotherapy for chronic depression. J Clin Psychol.

[13] Silva de Lima, M, Moncrieff J, Soares B. Drugs versus placebo for dysthymia. Cochrane Database Sys Rev. 2005; doi:10.1002/14651858.CD001130. 10.1002/14651858.CD001130.pub2 PMC10775756 26087170

[14] Michalak EE, Lam RW (2002). Breaking the myths: new treatment approaches for chronic depression. Can J Psychiatry.

[15] Cuijpers P, van Straten A, Schuurmans J, van Oppen P, Hollon DS, Andersson G (2010). Psychotherapy for chronic major depression and dysthymia: a meta-analysis. Clin Psychol Rev.

[16] Wolff AV, Hӧlzel LP, Westphal A, Härter M, Kriston L (2012). Combination of pharmacotherapy and psychotherapy in the treatment of chronic depression: a systematic review and meta-analysis. BMC Psychiatry.

[17] Spijker J, van Straten A, Bockting CL, Meeuwissen JA, van Balkom AJ (2013). Psychotherapy, antidepressants and their combination for chronic major depressive disorder: a systematic review. Can J Psychiatry.

[18] Kriston L, von Wolff A, Westphal S, Holzel LP, Harter M (2014). Efficacy and acceptability of acute treatments for persistent depressive disorder: a network meta-analysis. Depress Anxiety.

[19] Jobst A, Brakemeier E-L, Buchheim A, Caspar F, Cuijpers P, Ebmeier KP, Falkai P, Jan van der Gaag R, Gaebel W, Herpertz S, Kurimay T, Sabaß L, Schnell K, Schramm E, Torrent C, Wasserman D, Wiersma J, Padberg F (2016). European Psychiatric Association guidance on psychotherapy in chronic depression across Europe. Eur Psychiatry.

[20] Fonagy P, Rost F, Carlyle JA, McPherson S, Thomas R, Pasco Fearon RM, Goldberg D, Taylor D (2015). Pragmatic randomized controlled trial of long-term psychoanalytic psychotherapy for treatment-resistant depression: the Tavistock Adult Depression Study (TADS). World Psychiatry.

[21] Dougherty LR, Klein DN, Davila J (2004). A growth curve analysis of the course of dysthymic disorder: the effects of chronic stress and moderation by adverse parent-child relationships and family history. J Consult Clin Psychol.

[22] Durbin CE, Klein DN, Schwartz JE (2000). Predicting the 2 ½-year outcome of dysthymic disorder: the roles of childhood adversity and family history of psychopathology. J Consult Clin Psychol.

[23] Klein DN, Arnow BA, Barkin JL, Dowling F, Kocsis JH, Leon AC, Manber R, Rothbaum BO, Trivedi MH, Wisniewski SR (2009). Early adversity in chronic depression: clinical correlates and response to pharmacotherapy. Depress Anxiety.

[24] Klein DN, Santiago NJ (2003). Dysthymia and chronic depression: introduction, classification, risk factors and course. J Clin Psychol.

[25] Wiersma JE, Hovens JG, van Oppen P, Giltay EJ, van Schaik DJ, Beekman AT (2009). The importance of childhood trauma and childhood life events for chronicity of depression in adults. J Clin Psychiatry.

[26] Teicher MH, Samson JA (2013). Childhood maltreatment and psychopathology: a case for ecophenotypic variants as clinically and neurobiologically distinct subtypes. Am J Psychiatry.

[27] Angst J, Gamma A, Rossler W, Ajdacic V, Klein DN (2011). Childhood adversity and chronicity of mood disorders. Eur Arch Psychiatry Clin Neurosci.

[28] Chu DA, Williams LM, Harris AW, Bryant RA, Gatt JM (2013). Early life trauma predicts self-reported levels of depressive and anxiety symptoms in nonclinical community adults: relative contributions of early life stressor types and adult trauma exposure. J Psychiatr Res.

[29] Lizardi H, Klein DN, Ouimette PC, Riso LP, Anderson RL, Donaldson SK (1995). Reports of the childhood home environment in early-onset dysthymia and episodic major depression. J Abnorm Psychol.

[30] Erkkilä J, Punkanen M, Fachner J, Ala-Ruona E, Pöntiö I, Tervaniemi M, Vanhala M, Gold C (2011). Individual music therapy for depression: randomised controlled trial. Br J Psychiatry.

[31] Mӧssler K, Chen X, Heldal TO, Gold C (2011). Music therapy for people with schizophrenia and schizophrenia-like disorders. Cochrane Database Syst Rev.

[32] Talwar N, Crawford MJ, Maratos A, Nur U, McDermott O, Procter S (2006). Music therapy for in-patients with schizophrenia: exploratory randomised controlled trial. Br J Psychiatry.

[33] Gold C, Mӧssler K, Grocke D, Heldal TO, Tjemsland L, Aarre T, Aarø LE, Rittmannsberger H, Stige B, Assmus J, Rolvsjord R (2013). Individual music therapy for mental health care clients with low therapy motivation: multicentre randomised controlled trial. Psychoth Psychosom.

[34] Gold C, Solli HP, Krüger V, Lie SA (2009). Dose-response relationship in music therapy for people with serious mental disorders: Systematic review and meta-analysis. Clin Psychol Rev.

[35] Maratos A, Gold C, Wang X, Crawford M. Music therapy for depression. Cochrane Database Syst Rev. 2008. doi:10.1002/14651858.CD004517.pub2. 10.1002/14651858.CD004517.pub2 18254052

[36] Albornoz Y. The effects of group improvisational music therapy on depression in adolescents and adults with substance abuse: a randomized controlled trial. Nordic J Music Ther. 2011. doi:10.1080/08098131.2010.522717.

[37] Grocke D, Bloch S, Castle D, Thompson G, Newton R, Stewart S, Gold C. Group music therapy for severe mental illness: a randomized embedded-experimental mixed methods study. Acta Psychiatr Scand. 2013:130:144–153. doi:10.1111/acps.12224. 10.1111/acps.12224 24256453

[38] Carr C, d’Ardenne P, Sloboda A, Scott C, Wang D, Priebe S (2012). Group music therapy for patients with persistent post-traumatic stress disorder- an exploratory randomized controlled trial with mixed methods evaluation. Psychol Psychother.

[39] Ansdell G (2014). How music helps in music therapy and everyday life.

[40] Rolvsjord R (2010). Resource oriented music therapy in mental health care.

[41] Slade M (2009). Personal recovery and mental illness. A guide for mental health professionals.

[42] Solli HP, Rolvsjord R, Borg M (2013). Toward understanding music therapy as a recovery-oriented practice within mental health care: a meta-synthesis of service users’ experiences. J Music Ther.

[43] Seligman MEP (1975). Helplessness: on depression, development and death.

[44] Erkkilä J, Ala-Ruona E, Punkanen M, Fachner J, Hargreaves D, Miell D, Macdonald R (2012). Creativity in improvisational psychodynamic music therapy. Musical imaginations: multidisciplinary perspectives on creativity, performance and perception.

[45] Elliott A (1999). Approach and avoidance motivation and achievement goals. Educ Psychol.

[46] Stern D (2010). Forms of vitality: Exploring dynamic experience in psychology, the arts, psychotherapy and development.

[47] Grocke D, Bloch S, Castle D, Stewart S, Richards L (2011). “Songs for life”: A group songwriting project for people with mental illness.

[48] Rolvsjord R, Gold C, Stige B (2005). Research rigour and therapeutic flexibility: Rationale for a therapy manual developed for a randomized controlled trial. Nordic J Music Ther.

[49] Cross I, Morley I, Malloch S, Trevarthen C (2009). The evolution of music: theories, definitions and the nature of the evidence. Communicative musicality: exploring the basis of human companionship.

[50] Kreutz G (2014). Singing and social bonding - introduction. Music Med.

[51] Wigram T, Elefant C, Malloch S, Trevarthen C (2009). Therapeutic dialogues in music: nurturing musicality of communication in children with autistic spectrum disorder and Rett syndrome. Communicative musicality: exploring the basis of human companionship.

[52] Keeler JR, Roth EA, Neuser BL, Spitsbergen JM, Waters DJM, Vianney J-M. The neurochemistry and social flow of singing: bonding and oxytocin. Front Hum Neurosci. 2015;9:518. doi:10.3389/fnhum.2015.00518. 10.3389/fnhum.2015.00518 PMC4585277 26441614

[53] Carr C. Modelling of intensive group music therapy for acute adult psychiatric inpatients. PhD thesis. Queen Mary University of London,Barts and the London School of Medicine and Dentistry; 2014.

[54] Baker F, Wigram T (2005). Songwriting: Methods, techniques and clinical applications for music therapy clinicians, educators and students.

[55] Baker F, Ballantyne J. You’ve got to accentuate the positive: Group songwriting to promote a life of enjoyment, engagement and meaning in older Australians. Nordic J Music Ther. 2013. doi:10.1080/08098131.2012.678372.

[56] Baker F (2013). Music therapists’ perceptions of the impact of group factors on the therapeutic songwriting process. Music Ther Persp.

[57] Craig P, Dieppe P, Macintyre S, Michie S, Nazareth I, Petticrew M. Developing and evaluating complex interventions: new guidance. Med Res Counc. 2006. http://www.mrc.ac.uk/documents/pdf/complex-interventions-guidance. Accessed 08 Nov 2016.

[58] Bird VJ, Le Boutillier C, Leamy M, Williams J, Bradstreet S, Slade M (2014). Evaluating the feasibility of complex interventions in mental health services: standardised measure and reporting guidelines. Br J Psychiatr.

[59] Lancaster GA, Dodd S, Williamson PR (2004). Design and analysis of pilot studies: recommendations for good practice. J Eval Clin Pract.

[60] Gold C, Erkillä J, Crawford M (2012). Shifting effects in randomised controlled trials of complex interventions: a new kind of performance bias?. Acta Psychiatr Scand.

[61] Thabane L, Ma J, Chu R, Cheng J, Ismaila A, Rios LP, Robson R, Thabane M, Giangregorio L, Goldsmith CH (2010). A tutorial on pilot studies: the what, why and how. BMC Med Res Method.

[62] Montgomery SA, Åsberg M (1979). A new depression scale designed to be sensitive to change. Br J Psychiatr.

[63] Hoffman TC, Glasziou PP, Boutron I, Milne R, Perera R, Moher D (2014). Better reporting of interventions: template for intervention description and replication (TiDieR) checklist and guide. BMJ.

[64] Chan A-W, Tetzlaff JM, Altman DG, Laupacis A, Gøtzsche PC, Krležla-Jerić K (2013). SPIRIT 2013 statement: defining standard protocol items for clinical trials. Ann Intern Med.

[65] Brown J. Experimental Design Generator and Randomiser (EDGAR-II). 2005. http://www.edgarweb.org.uk/. Accessed 08 Nov 2016.

[66] Williams JBW, Kobak KA (2008). Development and reliability of a structured interview guide for the Montgomery-Åsberg depression rating scale (SIGMA). Br J Psychiatr.

[67] Galinowski A, Lehert P (1995). Structural validity of MADRS during antidepressant treatment. Int Clin Psychopharmacol.

[68] Parker G, Rosen A, Emdur N, Hadzi-Pavlov D (1991). The life skills profile: psychometric properties of a measure assessing function and disability in schizophrenia. Acta Psychiatr Scand.

[69] Beck AT, Steer RA, Brown GK (1996). Manual for the Beck Depression Inventory-II.

[70] Steer RA, Ball R, Ranieri WF, Beck AT (1999). Dimensions of the Beck Depression Inventory-II in clinical depressed outpatients. J Clin Psychol.

[71] Dozois DJA, Dobson KS, Ahnberg MJL (1998). A psychometric evaluation of the Beck Depression Inventory-II. Psychol Assessment.

[72] Derogatis L, Melisaratos N (1983). The brief symptom inventory: an introductory report. Psychol Med.

[73] Rosenberg M (1965). Society and the adolescent self-image.

[74] Gray-Little B, Williams VS, Hancock TD (1997). An item response theory analysis of the Rosenberg Self-esteem scale. Pers Soc Psychol B.

[75] Robins RW, Hendin HM, Trzesniewski KH (2001). Measuring global self-esteem: construct validation of a single-item measure and the Rosenberg Self-esteem scale. Pers Soc Psychol B.

[76] Schwarzer R, Jerusalem M, Weinman J, Wright S, Johnston M (1995). Generalized self-efficacy scale. Measures in health psychology: a user’s portfolio.

[77] Luzczynska A, Sholz U, Schwarzer R. The general self-efficacy scale: multicultural validation studies. J Psychol. 2005. doi:10.3200/JRLP.139.5.439-457. 10.3200/JRLP.139.5.439-457 16285214

[78] Leganger A, Kraft P, Røysamb E (2000). Perceived self-efficacy in health behaviour research: Conceptualisation, measurement and correlates. Psychol Health.

[79] Atkisson C, Greenfield TK, Sederer LL, Dickey B (1996). The client satisfaction questionnaire (CSQ) scales and the service satisfaction scale-30 (SSS-30). Outcome assessment in clinical practice.

[80] Mundt JC, Marks IM, Shear K, Greist JH (2002). The work and social adjustment scale: a simple measure of impairment in function. Br J Psychiatry.

[81] Priebe S, Huxley S, Knight S, Evans S (1999). Application and results of the Manchester Short Assessment of Quality of Life (MANSA). Int J Soc Psychiatry.

[82] Priebe S, Huxley P, Knight S, Evans S, Priebe S, Loiver J, Kaiser W (2000). The Manchester Short Assessment of Quality of Life (MANSA). Quality of life and mental health care.

[83] Beecham JK, Knapp MRJ, Thornicroft G, Brewin C, Wing JK (1992). Costing psychiatric interventions. Measuring mental health needs.

[84] Chisholm D, Knapp MRJ, Knudsen HC, Amaddeo F, Gaite L, van Wijngaarden B (2000). Client socio-demographic and service receipt inventory- European version: development of an instrument for international research. Br J Psychiatry.

[85] Huelsman TJ, Nemanick RC, Munz DC (1998). Scales to measure four dimensions of dispositional mood: positive energy, tiredness, negative activation and relaxation. Educ Psychol Meas.

[86] Huelsman TJ, Furr RM, Nemanick JC (2003). Measurement of dispositional affect: construct validity and convergence with a circumplex model of affect. Educ Psychol Meas.

[87] Burns DD (1993). Ten days to self-esteem.

[88] Elliott R. Client change interview protocol. Network for research on experiential psychotherapies. http://experiential-researchers.org/instruments/elliott/changei.html. Accessed on 08 Nov 2016.

[89] Elliott R, Slatick E, Urman M, Frommer J, Rennie DL (2006). Qualitative change process research on psychotherapy: Alternative strategies. Qualitative psychotherapy research - methods and methodology.

[90] Sim J, Lewis M (2012). The size of a pilot study for a clinical trial should be calculated in relation to considerations of precision and efficiency. J Clin Epidemiol.

[91] Julious SA (2005). Sample size of 12 per group rule of thumb for a pilot study. Pharm Stat.

[92] Harris T, Brown GW, Robinson R (1999). Befriending as an intervention for chronic depression among women in an inner city. 1. Randomised controlled trial. Br J Psychiatry.

[93] Kocsis JH, Gelenberg AJ, Rothbaum BO, Klein DN, Trivedi MH, Manber R, Keller MB, Leon AC, Wisniewski SR, Arnow BA, Markowitz JC, Thase ME (2009). Cognitive behavioural analysis system of psychotherapy and brief supportive psychotherapy for augmentation of antidepressant nonresponse in chronic depression. The REVAMP trial. Arch Gen Psychiatry.

[94] Simpson S, Corney R, Fitzgerald P, Beecham J. A randomised controlled trial to evaluate the effectiveness and cost-effectiveness of counselling patients with chronic depression. Health Technol Assess. 2000;4(36):1–83. doi:10.3310/hta4360. 11134918

[95] Gold C, Rolvsjord R, Mӧssler K, Stige B. Reliability and validity of a scale to measure interest in music among clients in mental health care. Psychol Music. 2013; doi:10.1177/0305735612441739.

